# Antioxidant and Hepatoprotective Effects of the Red Ginseng Essential Oil in H_2_O_2_-Treated HepG2 Cells and CCl_4_-Treated Mice

**DOI:** 10.3390/ijms13022314

**Published:** 2012-02-21

**Authors:** Min-Ji Bak, Mira Jun, Woo-Sik Jeong

**Affiliations:** 1 Institute for Phytochemical-Drug Interactions, Department of Food & Life Sciences, College of Biomedical Science & Engineering, Inje University, Gimhae 621-749, Korea; E-Mail: redapplemj@hanmail.net; 2 Department of Food Science & Nutrition, Dong-A University, Busan 604-714, Korea; E-Mail: mjun@dau.ac.kr

**Keywords:** antioxidant enzymes, SOD, GPx, Catalase, MAPK, red ginseng essential oil, *Panax ginseng*

## Abstract

The aim of this study was to evaluate the antioxidant mechanisms of red ginseng essential oil (REO) in cells as well as in an animal model. REO was prepared by a supercritical CO_2_ extraction of waste-products generated after hot water extraction of red ginseng. In HepG2 cells, REO diminished the H_2_O_2_-mediated oxidative stress and also restored both the activity and expression of antioxidant enzymes such as superoxide dismutase, catalase and glutathione peroxidase. Administration of REO inhibited the phosphorylation of upstream mitogen-activated protein kinases (MAPKs) such as c-Jun *N*-terminal kinase, extracellular signal-regulated kinase, and p38. In mice, the CCl_4_-mediated elevation of serum aspartate transaminase and alanine transaminase as well as the induction of hepatic lipid peroxidation were decreased by REO administration. REO treatments also resulted in up-regulation of the antioxidant enzyme expression in the liver. Moreover, increased phosphorylations of MAPKs were inhibited after REO administration. Overall, REO seems to protect the liver from oxidative stress through the activation and induction of antioxidant enzymes via inhibition of MAPKs pathways.

## 1. Introduction

Oxidative stress is an important contributory factor to the pathophysiology of a variety of pathological conditions including cardiovascular dysfunctions, atherosclerosis, inflammation, carcinogenesis, reperfusion injury and neurodegenerative diseases [1]. Oxidative stress is defined as an increase in reactive oxygen species (ROS) and/or a decrease in the antioxidant defense mechanisms [2]. ROS include free radicals such as superoxide (O_2_^•−^), hydroxyl radical (•OH), peroxyl radical (RO_2_^•^) as well as non radical species such as hydrogen peroxide (H_2_O_2_). These ROS are created as part of the body’s normal metabolic process as well as by exogenous stimuli such as heavy metals and other toxicants. Increased generation of ROS and free radicals has been shown to occur in various disease conditions [3].

The effects of ROS/free radicals can be balanced by endogenous antioxidant enzymes including superoxide dismutase (SOD), catalase (CAT), glutathione dependent enzymes such as glutathione peroxidase (GPx), glutathione reductase (GR) as well as by antioxidant compounds such as ascorbic acid, α-tocopherol, glutathione and other dietary antioxidants, which scavenge radicals or neutralize ROS, thus maintaining redox balance [4]. Therefore, the antioxidant enzymes and antioxidant molecules are believed to play an important role in the prevention of oxidative stress-related diseases such as cancer, cardiovascular disease, Alzheimer’s disease and muscular degeneration [5]. The activity and expression of the antioxidant enzymes can be modulated by cellular proteins like mitogen-activated protein kinases (MAPKs) such as c-Jun *N*-terminal kinase (JNK), extracellular-regulated kinase (ERK), and p38, as well as the upstream kinases including phosphoinositide kinase-3 (PI3K)/Akt [6]. A variety of phytochemicals from fruits, vegetables and herbs have been studied for their ability to protect cells against oxidative stress via the induction or activation of antioxidant enzymes [7–10].

Ginseng (*Panax ginseng*) in Korea is classified into three categories comprising fresh, white and red ginseng [11]. Red ginseng is made by steaming fresh ginseng followed by a drying process and is the most widely consumed ginseng product in Korea. Red ginseng has been shown to have various pharmacological properties such as anti-diabetic [12], antioxidant [13,14], anticarcinogenic [14], anti-aging [15], and anti-obesity effects [12,16]. However, these beneficial effects of red ginseng have been studied mostly with its water-soluble fractions, probably because red ginseng is widely consumed in the form of hot-water extract or its concentrates.

Plant-derived essential oils and lipid-soluble bioactive compounds have gained attention for biological roles due to their higher bioavailability compared to water-soluble bioactive compounds. Recently, some essential oils extracted from plants have been reported to have antioxidant effects through scavenging radicals and inducing antioxidant enzymes [17–20]. Essential oil isolated from fresh ginseng has been shown to exert anti-obesity effects in a mouse model fed on a high-fat diet through the suppression of dietary TG absorption and/or regulation of PPAR-gamma expression [21]. Moreover, a recent study using hexane extract of red ginseng has shown a growth inhibition of human lung tumor xenografts in nude mice [22].

To elucidate the antioxidant activity and the underlying molecular mechanisms of lipophilic moiety of red ginseng, we prepared a red ginseng essential oil (REO) using a supercritical CO_2_ extraction and evaluated its effects on ROS formation, both on activity and expression of antioxidant enzymes as well as on the upstream kinases including MAPKs/Akt pathways, which were studied in cells and a mouse model.

## 2. Results and Discussion

### 2.1. Inhibition of ROS Production in HepG2 Cells

**Figure 1:**
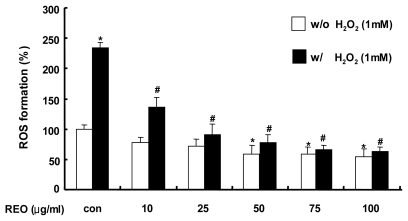
Effect of red ginseng essential oil (REO) on H_2_O_2_-induced reactive oxygen species (ROS) formation in HepG2 cells pre-treated with 2′,7′-dichlorofluorescin diacetate (DCF-DA) for 1 h and exposed to REO for another 1 h with or without H_2_O_2_. Values are means of three independent experiments ± S.D. (*n* = 3). * *p* < 0.05, significantly different from the vehicle control in the absence of H_2_O_2_. ^#^ *p* < 0.05, significantly different from the vehicle control in the presence of H_2_O_2_.

The oxidative damage caused by ROS may generate various diseases in humans such as aging, arthritis, cancer, inflammation, and heart diseases [23,24]. In the present study, 2′,7′-dichlorofluorescin diacetate (DCF-DA) staining was applied to examine whether REO was able to inhibit ROS production in H_2_O_2_-treated HepG2 cells. Hydrogen peroxide (H_2_O_2_) is produced endogenously from microsomes and peroxisomes and also by several physiological processes such as inflammatory respiratory burst and oxidative phosphorylation [6,25]. H_2_O_2_ has often been used as a model to investigate the mechanism of cell injury by oxidative stress [26,27]. When HepG2 cells were challenged with 1 mM H_2_O_2_, ROS were generated over 2-fold compared to the unchallenged control and the pretreatment with REO dose-dependently decreased the H_2_O_2_-mediated ROS formation (Figure 1). This dose-dependent antioxidant activity of REO was also observed even in the absence of H_2_O_2_. These results imply that REO may have an ability to directly scavenge ROS and/or free radicals.

### 2.2. Induction of Antioxidant Enzyme Activities in HepG2 Cells

**Figure 2:**
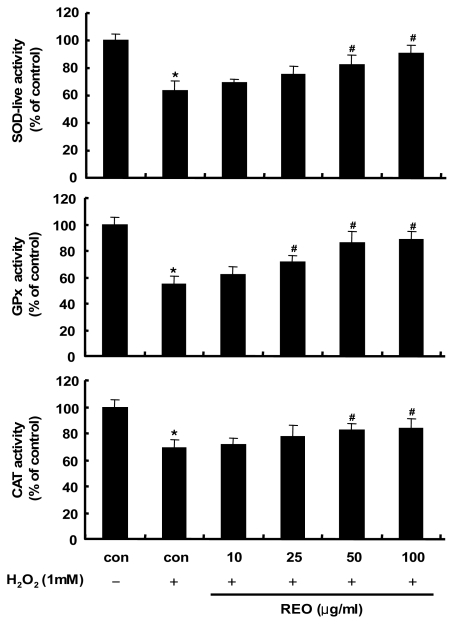
Effects of REO on superoxide dismutase (SOD)-like, glutathione peroxidase (GPx) and catalase (CAT) activities in H_2_O_2_-treated HepG2 cells. Data are mean ± SD values of three individual experiments. The values were compared with the control using analysis of variance followed by unpaired student’s *t* tests. * *p* < 0.05, significant differences from the unstimulated control group. # *p* < 0.05, significant differences from the H_2_O_2_-treated group.

In order to investigate whether these antioxidant properties of REO are related to the activity induction of antioxidant enzymes, HepG2 cells were treated with REO and the activities of antioxidant enzymes including SOD, GPx and CAT were measured. These enzymes are regarded as the first line of the antioxidant defense system against ROS generated during oxidative stress [28]. As shown in Figure 2, the activities of the three antioxidant enzymes were dramatically down-regulated by H_2_O_2_ (1 mM) treatment, while treatment with REO restored the enzymes activities in a dose dependent manner. Therefore, the REO appears to have antioxidant properties by stimulating the activity of endogenous antioxidant enzymes as well as by directly scavenging ROS/free radicals. Recently, a water-soluble ginsenoside Rb1 was reported to prevent H_2_O_2_-induced HUVECs senescence through upregulating endogenous antioxidants like SOD and decreasing vascular lipid peroxidation [29]. This study affirmed for the first time the antioxidant properties of the essential oil of red ginseng byproducts.

### 2.3. Induction of Antioxidant Enzyme Expressions in HepG2 Cells

**Figure 3:**
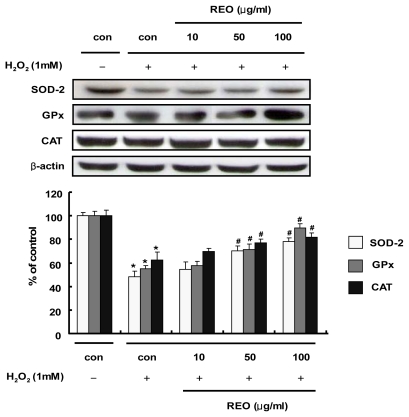
Effects of REO on SOD, GPx and CAT protein expressions in HepG2 cells. The bands shown are representative of three independent experiments with similar results. Values are means ± SD; *n* = 3. * *p* < 0.05, significantly different from the unstimulated control group. ^#^ *p* < 0.05, significantly different from the H_2_O_2_-treated group.

In addition to the enzyme activity stimulation by REO, the effects of REO on the protein expression of these antioxidant enzymes were evaluated in HepG2 cells. When cells were treated with H_2_O_2_ (1 mM) alone, the protein expressions of SOD, GPx and CAT were significantly diminished compared to the vehicle-treated control (Figure 3). However, the treatment with REO potently and dose-dependently re-induced the expression of the antioxidant enzymes. Accordingly, the REO seems to induce both activity and expression of the antioxidant enzymes. An aqueous extract of ginseng has been reported to restore the expression of these antioxidant enzymes in H_2_O_2_-injured primary cultures of rat astrocytes [30]. Ginseng saponins are also protective against alcohol-induced hepatic injury in mice by up-regulating the expression of these antioxidant enzymes [31]. Our data strongly imply that essential oil from red ginseng byproducts, but neither the water-soluble extract nor saponins, may participate in cellular protection not only directly as an antioxidant molecule (as shown in Figure 1) but also indirectly as a stimulator of antioxidant enzymes.

### 2.4. Effects of REO on Phosphorylations of Upstream Kinases in HepG2 Cells

**Figure 4:**
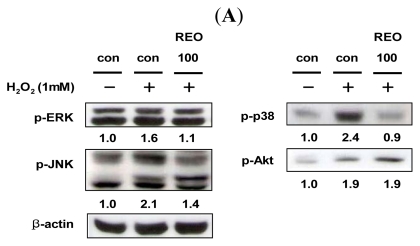

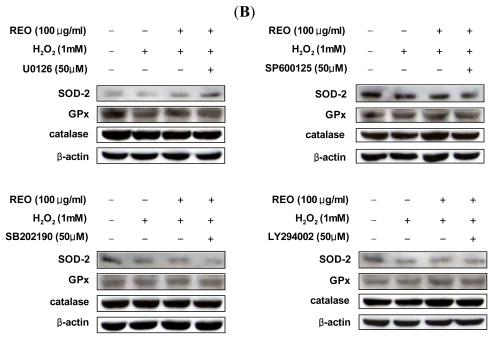
Effects of REO on mitogen-activated protein kinase (MAPK) and Akt phosphorylations. (**A**) HepG2 cells pretreated with vehicle or REO (100 μg/mL) before being incubated with H_2_O_2_ (1 mM) for 1 h. Then, the phosphorylations of target proteins were detected by Western blotting; (**B**) HepG2 cells pretreated with vehicle or 50 μM inhibitors against MAPK/ERK kinase (MEK) 1/2 (U0126), c-Jun *N*-terminal kinase (JNK) (SP600125), p38 (SB202190), and serine/threonine protein kinase Akt (LY294002) for 1 h prior to treatment with 100 μg/mL REO for 1 h. The bands shown are representatives of three independent experiments with similar results.

The MAPK family plays important roles in regulation of cell proliferation and cell death in response to various cellular stresses [32]. To determine whether REO can modulate the upstream signaling pathways, HepG2 cells were treated with REO and the phosphorylations of ERK, JNK, p38 and Akt were analyzed. The results indicated that H_2_O_2_ stimulated phosphorylations of all MAPKs and Akt (Figures 4A). The induced expression of phosphorylated ERK, JNK and p38 by H_2_O_2_ was down-regulated by REO, whereas phosphorylation of Akt was up-regulated (Figure 4A). To further elucidate the modulatory effects of REO on MAPK and Akt signaling pathways and their involvement in antioxidant enzyme expression, cells were treated with specific inhibitors of each MAPKs and Akt (U0126, an inhibitor of MEK1/2; SP600125, an inhibitor of JNK; SB202190, an inhibitor of p38; and LY294002, an inhibitor of phosphoinositide 3-kinase (PI3K)/Akt). The stimulated expression of SOD by REO was down-regulated by treatment with the p38-specific inhibitor, and those of GPx and CAT by REO were decreased by treatments with JNK specific inhibitor (Figure 4B). Accordingly, REO seems to induce the expression of SOD through inhibition of p38 phosphorylation, while it induces the expression of GPx and CAT via blockade of JNK phosphorylation. Zhang and Wang [33] have reported that a notoginseng saponin notoginsenoside R1 inhibits TNF-alpha-induced ERK activation in human arterial smooth muscle cells by suppressing NADPH oxidase-mediated ROS generation and directly scavenging ROS. Notoginseng n-butanol extracts have been shown to inhibit the LPS-stimulated activation of JNK and ERK signaling in RAW264.7 cells [34]. So far, there have been few reports on the effect of ginseng ingredients on the relationship between antioxidant enzymes and MAPKs pathways. Our previous study demonstrated that a flavonoid quercitrin from *Cedrela sinensis* down-regulates H_2_O_2_-induced phosphorylations of Akt and three MAPKs (ERK, JNK, and p38) in HepG2 cells [7]. In the study, we found that quercitrin-induced expression of GPx was ameliorated by the p38-specific inhibitor and the Akt/PI3K inhibitor, whereas the expression of SOD by quercitrin was diminished only by the p38 inhibitor. Upstream molecules such as MAPKs and Akt are certainly involved in modulation of the expression and activity of the antioxidant enzymes. However, the mode of regulation seems to be different by cell types as well as by compounds treated.

### 2.5. Effects of REO on Serum ALT and AST Activity in Mice

Based on the antioxidant activity of REO at cellular level, the effect of REO was further examined in an animal model. Male Balb/c mice were treated with either REO (10 and 50 mg/kg b.w.) or silymarin (50 mg/kg b.w.), a known hepatoprotective phytochemical as a positive control, and challenged with CCl_4_ (carbon tetrachloride, dissolved in corn oil (20% v/v, 3 mL/kg b.w.)) for three days per week for four weeks. CCl_4_ is often used in experimental animal models of severe hepatic damage by generation of oxidative stress and activation of immune cells, which can lead to architectural and functional alteration [35].

**Figure 5:**
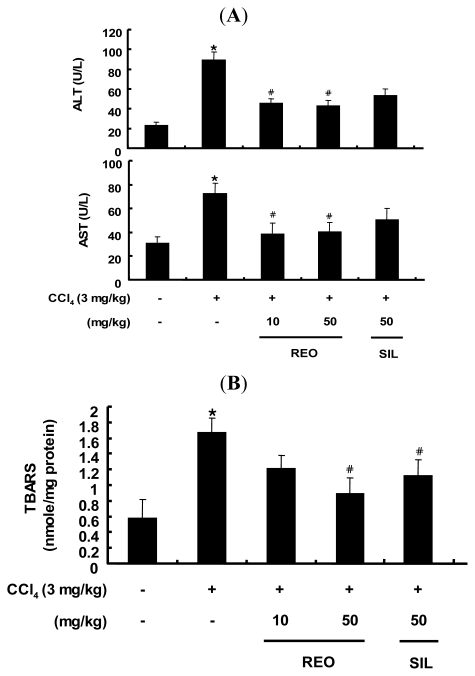
Effects of REO administration on the activities of (**A**) serum alanine transaminase, serum aspartate transaminase and (**B**) hepatic thiobarbituric acid reactive Substance (TBARS) content in CCl_4_-treated mice. Each value represents the mean ± SD for 8 mice. * *p* < 0.05, significantly different from the unstimulated control group. ^#^ *p* < 0.05, significant differences from the CCl_4_-treated group.

As shown in Figure 5A, the administration of CCl_4_ for 4 weeks dramatically elevated the serum levels of routine liver markers of aspartate transaminase (AST) and alanine transaminase (ALT) compared to the vehicle-treated normal control group, indicating significant hepatotoxicity of CCl_4_ treatment. In contrast, administration of REO at both 10 and 50 mg/kg b.w. significantly restored ALT and AST levels in CCl_4_-treated mice. Silymarin at 50 mg/kg b.w. also decreased the levels of ALT and AST but not as effectively as REO in attenuation of hepatotoxicity caused by CCl_4_. There have been several animal studies on the hepatoprotective effects of ginseng extracts and saponins against exposure to toxicants such as CCl_4_ [36], benzo[alpha]pyrene [37], ethanol [38] and tert-butyl hdroperoxide [39]. Yet, the current study affirmed for the first time the hepatoprotective activity of the essential oil of red ginseng byproducts.

### 2.6. Effect of REO on Hepatic TBARS Content in Mice

Lipid peroxidation is considered to be one of the principal results of CCl_4_-induced liver injury [40]. As a biomarker for lipid peroxidation, hepatic lipid peroxidation was determined by measuring the TBARS concentration in liver tissues in CCl_4_-treated mice. The hepatic TBARS concentrations were increased by CCl_4_ treatment, but these increases of lipid peroxidation were dose-dependently inhibited in both REO groups and silymarin group and the inhibition by REO was more potent than that by silymarin at the same dose of 50 mg/kg b.w. (Figure 5B). These data suggest that REO may exert its antioxidant effects through the inhibition of hepatic lipid peroxidation.

### 2.7. Effects of REO on the Activity and Expression of Hepatic Antioxidant Enzymes in Mice

**Figure 6:**
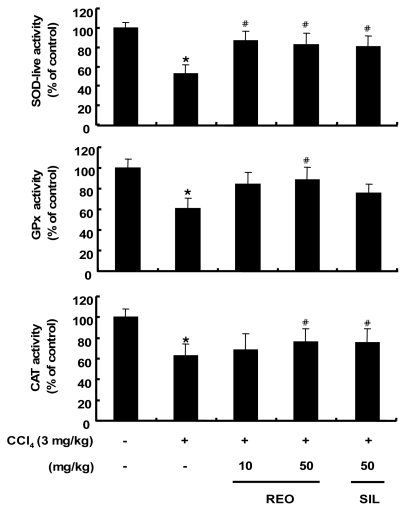
Effects of REO on hepatic SOD, GPx and CAT activities in CCl_4_-treated mice. Each value represents the mean ± SD for 8 mice. * *p* < 0.05, significantly different from the unstimulated control group. ^#^ *p* < 0.05, significant differences from the CCl_4_-treated group.

**Figure 7:**
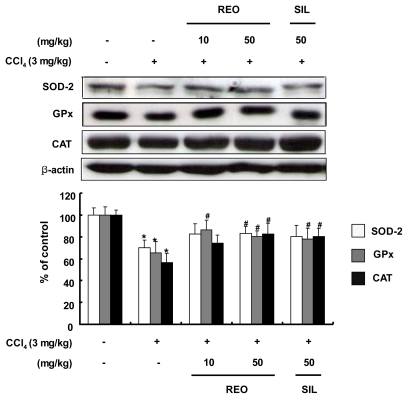
Effects of REO on SOD, GPx and CAT protein expression in CCl_4_-treated mice. Each value represents the mean ± SD for 8 mice. * *p* < 0.05, significant differences from the unstimulated control group. ^#^ *p* < 0.05, significant differences from the CCl_4_-treated group.

The administration of CCl_4_ is known to increase the production of ROS/free radicals, resulting in a significant reduction of the endogenous antioxidant enzymes activity [41]. The activities of the three hepatic antioxidant enzymes including SOD, GPx and CAT were evaluated in mice treated with CCl_4_ alone (Figure 6). However, administration of REO at both doses of 10 and 50 mg/kg b.w. resulted in significant increase in the SOD activity and similar results were observed in the silymarin-treated group (50 mg/kg b.w.). In GPx activity, only REO administration at 50 mg/kg b.w. resulted in a significant recovery. CAT activity increased significantly in both REO and silymarin groups at 50 mg/kg b.w. In addition to the activity of the hepatic antioxidant enzymes, the protein expression levels after exposure of mice to REO and CCl_4_ were evaluated by Western blotting analysis of liver tissue. Injection with CCl_4_ resulted in significant down-regulations of the protein expression levels of SOD, GPx and CAT compared with the normal mice control (Figure 7). However, administration of REO at 50 mg/kg b.w. significantly improved protein expressions of all three antioxidant enzymes in the liver of mice treated with CCl_4_. Therefore, REO seems to enhance both the activity and expression of hepatic antioxidant enzymes *in vivo* as well as *in vitro* cell lines. Earlier studies have reported that administration of ginsenosides increases the activities SOD, GPx and CAT in mice [42] Our results suggest that REO may attenuate oxidative stress by increasing both the activity and expression of the antioxidant enzymes *in vitro* as well as *in vivo*.

### 2.8. Effects of REO on Phosphorylations of Upstream Kinases in Mice

**Figure 8:**
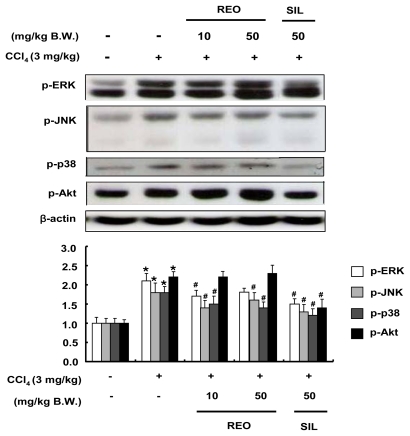
Effects of REO on MAP kinase and Akt protein expressions in liver of CCl_4_-treated mice. Each value represents the mean ± SD for 8 mice. * *p* < 0.05, significant differences from the unstimulated control group. ^#^ *p* < 0.05, significant differences from the CCl_4_-treated group.

During CCl_4_ challenge, oxidative stress activates MAP kinase, leading to phosphorylation of JNK and p38 [43]. On the other hand, ERK 1/2 is involved in survival signals by regulating cell proliferation after partial hepatectomy or CCl_4_ intoxication [44]. The effects of REO on the phosphorylation of hepatic upstream kinases such as MAPKs and Akt were analyzed to better understand the underlying mechanisms association between REO and the hepatic defense system. As shown in Figure 8, the phosphorylation levels of all three MAPKs and Akt were up-regulated (1.8–2.2 fold) by CCl_4_ treatment. Administration of REO resulted in inhibition of CCl_4_-mediated phosphorylations of hepatic MAPKs while it did not affect the phosphorylation of Akt. These results correspond with those observed in the cell line model. This study affirmed for the first time the involvement of upstream signaling pathways in hepatoprotective activity of the essential oil of red ginseng byproducts.

## 3. Experimental Section

### 3.1. Chemicals and Reagents

H_2_O_2_, nitro blue tetrazolium salt, xanthine, copper chloride, glutathione, xanthine oxidase from bovine milk (0.1–0.4 U/mg of protein), polyethylene glycol oil, silymarin, carbon tetrachloride and glutathione reductase from baker’s yeast (*Saccharimyces cerevisiae*) (100–300 U/mg of protein) were purchased from Sigma Chemical Co. (St. Louis, MO, USA). 2′,7′-dichlorodihydrofluorescein (DCF-DA) was purchased from Molecular Probe Inc. (Eugene, OR, USA). F-12 medium, fetal bovine serum and penicillin/streptomycin were obtained from Hyclone (Logan, UT, USA). Antibodies against SOD, CAT, GPx, phosphorylated ERK, phosphorylated JNK, phosphorylated p38, and phosphorylated Akt were purchased from Cell Signaling Technology Inc. (Beverly, MA, USA). Anti β-actin and horseradish peroxidase-conjugated anti-goat and anti-rabbit immunoglobulin IgG were purchased from Santa Cruz Biotechnology (Santa Cruz, CA, USA). Specific inhibitors against upstream targets, including U0126, SP600125, SB202190 and LY294002 were obtained from Cell Signaling Technology. All other reagents were of the highest quality generally available.

### 3.2. Preparation of REO

The supercritical CO_2_ extraction was performed using a pilot-scale automated equipment (Ilshin Autoclave Co., LTD, Daejeon, Korea). One hundred kilograms of red ginseng byproduct powder were placed into a high-pressure extraction tank and the supercritical CO_2_ extraction was conducted with a pressure of 450 bar at 65 °C. The CO_2_ was liquefied by a cooler and pressurized to operating pressure using a high pressure pump. The pressurized carbon dioxide was then heated to operating temperature and pumped into the extraction tank (cell) during the supercritical fluid extraction process. The essential oil and other lipophilic substances in the separator were collected via supercritical CO_2_ through a needle valve with heater for 3–4 h extraction times.

### 3.3. Cell Culture

HepG2 human hepatoma cells were obtained from American Type Culture Collections (Rockville, MD, USA). Cells were maintained in F-12 medium supplemented with 10% fetal bovine serum, 100 units/mL penicillin, 100 μg/mL streptomycin, 1% essential amino acids, 1% glutamax and 0.1% insulin, in a humidified atmosphere of 95%, 5% CO_2_ at 37 °C.

### 3.4. Animals

Male Balb/c mice, 6 weeks old (weighing 18–20 g), were purchased from Hyochang Science (Daegu, Korea) and were given a standard laboratory diet and distilled water *ad libitum* under a 12/12 h light/dark cycle in a temperature controlled room (22 ± 2 °C). The protocol for all animal experiments was approved by the Animal Care and Used Committee of Inje University. The animals were acclimatized for seven days before experiments were performed. The mice were divided into five groups (*n* = 8 per group) including: the normal control group and received polyethylene glycol oil (PEG) as vehicle for 5 weeks; the CCl_4_ group as a negative control received PEG for 1 week and then injected i.p. with CCl_4_ dissolved in corn oil (20% v/v, 3 mL/kg) three times per week for 4 weeks. REO groups were administered with 10 or 50 mg/kg of REO, *p.o.* three times per week and then treated with REO and CCl_4_ for another 4 weeks. The Silymarin group served as a positive control and was administered with 50 mg/kg of silymarin, *p.o.* three times per week and then treated with REO and CCl_4_ for another 4 weeks.

### 3.5. Assessment of ROS Production in Cells

The effect of REO on ROS production was evaluated in HepG2 cells using a cell permeable probe, DCF-DA [45]. The cells were seeded onto 96 well plates, grown until confluency and pre-incubated with DCF-DA for 1 h at 37 °C in darkness. After washing out the excess probe, the cells were treated with vehicle (0.1% DMSO) or REO in the presence or absence of 1 mM H_2_O_2_ for 12 h and then washed twice with 1× ice-cold PBS. The fluorescence was measured at 485/20 nm excitation and 528/20 nm emission in a fluorescence multi-detection reader (Synergy HT™ Multi-detecction microplate reader; BioTek Instruments Inc, VT, USA).

### 3.6. Assessment of CAT and GPx Activities in HepG2 Cells and Liver Tissues

HepG2 cells were seeded in a six-well plate until 80–90% confluency. After starvation for 12 h, the cells were treated with samples for 24 h and harvested in an ice-cold cell lysis buffer (pH 7.4). The cell debris was removed by centrifugation at 13,000 *g* for 10 min and the supernatant was used for further experiments. For the mouse model, liver tissue was homogenized in cell lysis buffer. The protein concentration for both cells and tissues was determined using the BCA protein assay (Pierce Biotechnology, Inc., Rockford, IL, USA) according to the manufacturer’s instructions. Each supernatant containing an equal amount of protein (10 μg) was used for the enzyme activity assay.

CAT activity was measured according to the method of Carrillo *et al*. [46]. One unit of CAT was defined as the amount of enzyme required to decompose 1.0 μM of H_2_O_2_ in 1 min. The reaction was initiated by the addition of freshly prepared 20 mM H_2_O_2_ (1 mL). The rate of decomposition of H_2_O_2_ was measured at 240 nm for 1 min. The enzyme activity was expressed as U/mg protein.

GPx activity was evaluated by the method of Bogdanska *et al*. [47]. The reaction mixture consisted of 0.1 M phosphate buffer (pH 7.0), 1 mM EDTA, 10 mM glutathione (GSH), 1 mM NaN_3_, 1 unit of glutathione reductase, 1.5 mM NADPH and cell lysates. The activity was calculated using the molar extinction coefficient for NADPH of 6.22 μmol/cm at 340 nm.

### 3.7. Assessment of SOD-Like Activity in HepG2 Cells and Liver Tissues

SOD-like activity was measured by the inhibition of nitro blue tetrazolium reduction due to the superoxide anion generated by the combination xanthine and xanthine oxidase [48]. The reaction mixture was buffered with 50 mM sodium carbonate buffer (pH 10.2) containing 3 mM xanthine, 0.75 mM nitro blue tetrazolium, 3 mM EDTA and 50 μL cell lysates. After pre-incubation for 30 min at room temperature, the reaction was initiated by adding 50 μL xanthine oxidase (0.1 mg/mL) and stopped by addition of 6 mM copper (II) chloride and centrifuged at 1500 *g* for 15 min. The absorbance of the reaction mixture was measured at 560 nm.

### 3.8. Western Blotting Analysis

The whole cell extracts for immunoblotting SOD, CAT and GPx were isolated from either H_2_O_2_-stimulated HepG2 cells or liver tissue from the CCl_4_-treated mice. These sample proteins were separated in a 10% polyacrylamide gel at 100 V and transferred onto polyvinylidene difluoride (PVDF) membranes (Millipore, Bedford, MA, USA) using a semi-dry transfer system. The membranes were blocked with 5% non-fat dry milk in 1× PBST buffer (0.1% Tween 20 in PBS) overnight at 4 °C and incubated with rabbit polyclonal antibody in PBST solution containing 5% non-fat dry milk. After washing 3 times with PBST, the membranes were incubated for 1 h at room temperature with anti-rabbit and anti-goat antibodies with horseradish peroxidase for 1 h at room temperature, and washed with PBST 3 times. Final detection was performed with enhanced chemiluminescence (ECL™) Western blotting reagents (Santa Cruz).

### 3.9. Assessment of Serum Marker Enzymes

The enzymatic activities of serum ALT and AST were used as biochemical markers for hepatotoxicity. Blood samples from mice were centrifuged at 3000 *g* at 4 °C for 10 min and the ALT and AST activities in serum supernatants were determined using commercially available kits (Youngdong Pharm, Seoul, Korea).

### 3.10. Assessment of Thiobarbituric Acid Reactive Substance (TBARS) in Tissue

Liver tissues were homogenized in an ice-cold lysis buffer and a portion of the homogenate was measured immediately for TBARS value using the method of Ohkawa *et al*. [49]. Malondialdehyde, an end product of lipid peroxidation, reacted with thiobarbituric acid. The absorbance of the resulting chromophore was measured at 535 nm.

### 3.11. Statistical Analysis

The data were expressed as mean ± SD values. The values were compared with the control using analysis of variance followed by unpaired Student’s t tests. A *P* value of <0.05 was considered significant.

## 4. Conclusions

Red ginseng has been extensively studied during recent decades and most of the studies have focused on the water soluble parts with ginsenoside saponins due to its normal consumption pattern. Results of this study clearly suggest that red ginseng oil has the ability to protect cells/tissues against oxidative damage by directly scavenging ROS, by inhibiting lipid peroxidation, as well as by inducing both activity and expression of cellular antioxidant enzymes activity and expression through inhibiting MAPK signaling pathways. Therefore, REO might be considered as a useful source of cellular defense agents at least in liver cells and/or tissues. However, studies on the active ingredients in REO should be followed up and will be reported later.
